# Three‐dimensional quantitative measurement of buccal augmented tissue with modified coronally advanced tunnel technique and de‐epithelialized gingival graft: a prospective case series

**DOI:** 10.1186/s12903-021-01522-2

**Published:** 2021-03-25

**Authors:** Fei Xue, Rui Zhang, Yu Cai, Yong Zhang, Ni Kang, Qingxian Luan

**Affiliations:** 1 Department of First Clinical Division, Peking University School and Hospital of Stomatology & National Clinical Research Center for Oral Diseases & National Engineering Laboratory for Digital and Material Technology of Stomatology & Beijing Key Laboratory of Digital Stomatology, 22 Zhongguancun South Avenue, Haidian District, Beijing, 100081 People’s Republic of China; 2 Central Laboratory, Peking University School and Hospital of Stomatology & National Clinical Research Center for Oral Diseases & National Engineering Laboratory for Digital and Material Technology of Stomatology & Beijing Key Laboratory of Digital Stomatology, 22 Zhongguancun South Avenue, Haidian District, Beijing, 100081 People’s Republic of China; 3 Department of Third Clinical Division, Peking University School and Hospital of Stomatology & National Clinical Research Center for Oral Diseases & National Engineering Laboratory for Digital and Material Technology of Stomatology & Beijing Key Laboratory of Digital Stomatology, 22 Zhongguancun South Avenue, Haidian District, Beijing, 100081 People’s Republic of China; 4 Department of Periodontology, Peking University School and Hospital of Stomatology & National Clinical Research Center for Oral Diseases & National Engineering Laboratory for Digital and Material Technology of Stomatology & Beijing Key Laboratory of Digital Stomatology, 22 Zhongguancun South Avenue, Haidian District, Beijing, 100081 People’s Republic of China

**Keywords:** Intraoral scanning, Digital measurement, Modified coronally advanced tunnel (MCAT), De‐epithelialized gingival graft (DGG), Periodontal plastic surgery

## Abstract

**Background:**

The aim of this study is to investigate three-dimensional quantitative analysis of buccal augmented tissue alterations after surgery using a modified coronally advanced tunnel (MCAT) technique combined with a de-epithelialized gingival graft (DGG) within 1 year post-op, based on intraoral scanning.

**Methods:**

25 Cairo class I gingival recession defects were treated using an MCAT technique with DGG. Digital impressions were taken using an intraoral scanner at baseline, 2 weeks, 6 weeks, 3 months, and 1 year after the surgery. Three-dimensional quantitative measurements within 1 year were analyzed for buccal augmented tissue after surgery, including postoperative gingival height gain (GHG), area gain (GAG), volume gain (GVG) and mean thickness (GMT) of region of interest, as well as the tissue thickness change at 1, 2, and 3 mm (TTC1, TTC2, and TTC3) apical to the cemento-enamel junction.

**Results:**

Postoperative GHG, GAG, GVG, and GMT were distinctly encountered at 2 weeks post-op, then gradually decreased. At 1 year, GHG, GAG, GVG, and GMT were 2.211 ± 0.717 mm, 7.614 ± 2.511 mm^2^, 7.690 ± 4.335 mm^3^ and 0.965 ± 0.372 mm, respectively. Significant decreases were recorded between 6 weeks and 1 year in terms of GHG, GAG, and GVG. The GMT was sustained after 6 weeks with an increase of nearly 1 mm at 1 year. TTC1 and TTC2 yielded thicker tissue change than TTC3.

**Conclusions:**

Three-dimensional quantitative measurements taken via intraoral scanning showed that buccal augmented tissue acquired via MCAT with DGG tends to be stable after 3 months post-op. Digital measurement can be applied in periodontal plastic surgery as a clinically feasible and non-invasive evaluation method for achieving volumetric outcomes.

*** Trial registration*:**

This study was retrospectively registered in the Chinese Clinical Trial Registry: ChiCTR1900026768. Date of registration: 21/10/2019.

## Background

Gingival recession is defined as the displacement of the gingival margin apical relative to the cemento-enamel junction (CEJ) [1] resulting in partial exposure of the root surface. It is a frequent clinical feature in both populations with both good and poor oral hygiene [2, 3]. Several predisposing factors, such as thin gingival phenotype, traumatic toothbrushing habit, cervical restorations, and orthodontic treatment, have been suggested as contributions to the development of gingival recession [4]. Among multiple surgical procedures [5], the modified coronally advanced tunnel (MCAT) technique in combination with de-epithelialized gingival graft (DGG), has been introduced to as a treatment to increase gingival dimensions and to cover the exposed root surface effectively and with long-term stability [6–8].

Objective measurement parameters such as gain in the height, width, and thickness of the gingiva, mean root coverage (MRC), complete root coverage (CRC) [9, 10], as well as subjective assessments such as the root coverage esthetic score (RES) [11] and the smile esthetic index (SEI), are commonly used to evaluate the outcome of periodontal plastic surgery. In terms of linear measurements, the most common instrument used is a periodontal probe in millimeter scale. As reliable a method as it is, it may be limited by the errors associated with rounded reading and interpretation angles. Other common methods involving plaster models also have a tendency to be inaccurate due to potential deformation in manufacturing and long-term storage. Transgingival probing, ultrasound, and radiographic methods are used frequently to assess gingival thickness [12, 13]. These methods all have unavoidable shortcomings such as invasiveness, tissue variance due to local anesthesia or compression, radiation exposure, and poor site repeatability. The heterogeneity of measurement methods also makes it difficult to compare data within the literature. Therefore, in order to determine the efficacy of the differing techniques and grafts utilized in periodontal plastic surgery, it may be beneficial to explore more convenient quantitative methods to evaluate minor changes in soft tissue morphology more precisely in the period following periodontal plastic surgery.

In recent years, digital measurement methods, using intraoral scanning instruments, have provided clinicians with a new choice [14]. By using analysis software, the measurements can be obtained repeatably from multiple angles [15]. Even volumetric alteration measurements can be obtained quantitatively [16]. In addition, the digital data can be stored for a long period of time, making it possible to conduct future secondary research or long-term follow-ups [17]. Therefore, the focus of this study was three-dimensional quantitative analysis of buccal augmented tissue after periodontal plastic surgery within 1 year based on intraoral scanning.

## Methods

### Study subjects

Patients were recruited from June 2019 to December 2020 in the Department of Periodontology, the First Clinical Division, Peking University Hospital and School of Stomatology. All procedures performed in the present study involving human participants were in accordance with the ethical standards of the institutional and/or national research committee and in accordance with the declaration of Helsinki 1975, which was revised in 2000. The Ethics Committee of Peking University Stomatology Hospital approved the study protocol (PKUSSIRB-201947089).Written informed consent for participation was obtained from each subject recruited in this study. This study has been registered in the Chinese Clinical Trial Registry (ChiCTR.1900026768).

Inclusion criteria were as follows: (1) 18–65 years old; (2) presented at least 2 mm Cairo class I gingival recession [18] at incisors, canines, and premolars; (3) detectable CEJ with no cervical caries or restorations; (4) bleeding index ≤ 1, probing depth ≤ 3 mm, keratinized tissue width (KTW) ≥ 2 mm; (5) full-mouth plaque score and full-mouth bleeding on probing ≤ 15%. Patients affected by systemic diseases, pregnant or breastfeeding women, and smokers were excluded from the study.

### Surgical procedures

**Fig. 1:**
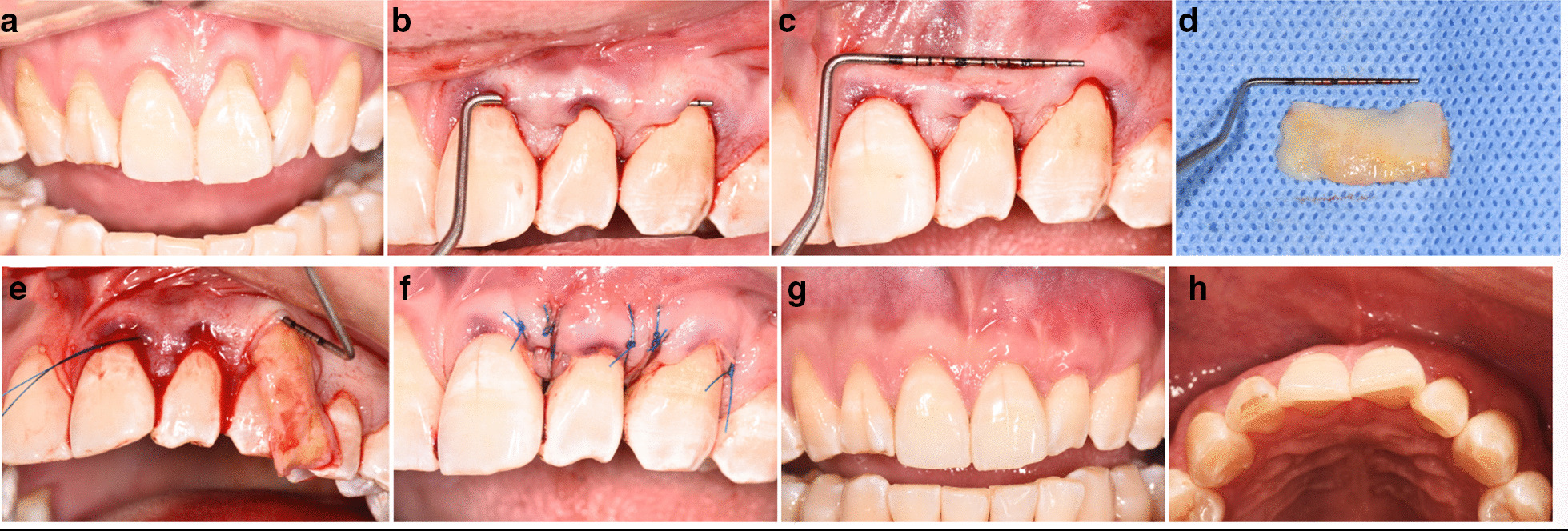
Surgical protocol of gingival recession defects treated using MCAT with DGG. Baseline (**a**); prepared tunnel (**b**, **c**); harvested DGG from palate (**d**); DGG inserted into the tunnel (**e**); the tunneled flap sling sutured coronally (**f**); facial and occlusive view at 6 weeks post-op (**g**, **h**)

All subjects were treated using an MCAT [19] technique in combination with DGG by the same surgeon (F.X.). The surgical procedure was as follows (Fig. 1): the exposed root was planed before the operation. After application of local anesthesia, the tunnel was prepared as a split thickness flap above the muco-gingival junction while retaining the connection between the buccal and lingual papillae.Subsequently, a 1 mm in thickness DGG was harvested from the palate and carefully inserted into the tunnel. Sling sutures were used to coronally reposition the tissue. Patients were instructed to rinse the mouth twice a day using 0.2% chlorhexidine solution for 2 weeks. The sutures were removed at 2 weeks post-op. Re-examinations were conducted at 1, 2, and 6 weeks and then 3, 6, and 12 months after the surgery. Supragingival plaque was removed when necessary.

### Study process

An intraoral scanner (3 shape Trios 2 pod color, 3shape, Denmark) was used to obtain digital models at baseline (BL), 2 weeks (2w), 6 weeks (6w), 3 months (3m), and 1 year (1y) after the surgery by the same researcher (Y.Z.). The scanning range included not only the surgically-treated tooth, but also at least two adjacent teeth on each side. Data were output in “*.stl” file format and imported into Geomagic Studio 2013 (Geomagic, Morrisvillle, USA) for measurement. The surfaces of the teeth, excluding the contact area, were invoked as the matching area and superimposed by utilizing “Best Fit Alignment”. This re-determined the mesial-distal direction as the X-axis, the buccal-lingual direction as the Y-axis, and the tooth’s longitudinal axis as the Z-axis.

### Digital measurement parameters in the region of interest (ROI)

- *Gingival recession height (GRH)* The vertical distance between the lowest point of preoperative gingival margins and the CEJ. (mm)
- *Gingival recession width (GRW)* The horizontal distance between the bilateral gingival margins at the height of the CEJ. (mm)
- *Root exposure area (REA)* The area bounded by the preoperative marginal gingival and the CEJ. (mm^2^)
- *Gingival height gain (GHG)* The vertical distance between the lowest points of the preoperative and postoperative gingival margins (the height of the ROI). (mm)
- *Gingival area gain (GAG)* The difference in area between the preoperative and postoperative gingival margins (the area of the ROI). (mm^2^)
- *Gingival volume gain (GVG)* The difference in volume above the GAG between the preoperative and postoperative model surfaces (the volume of the ROI). (mm^3^)
- *Gingival mean thickness (GMT)* The mean thickness of the gingival of covered area (the mean thickness of the ROI). (mm)

**Fig. 2:**
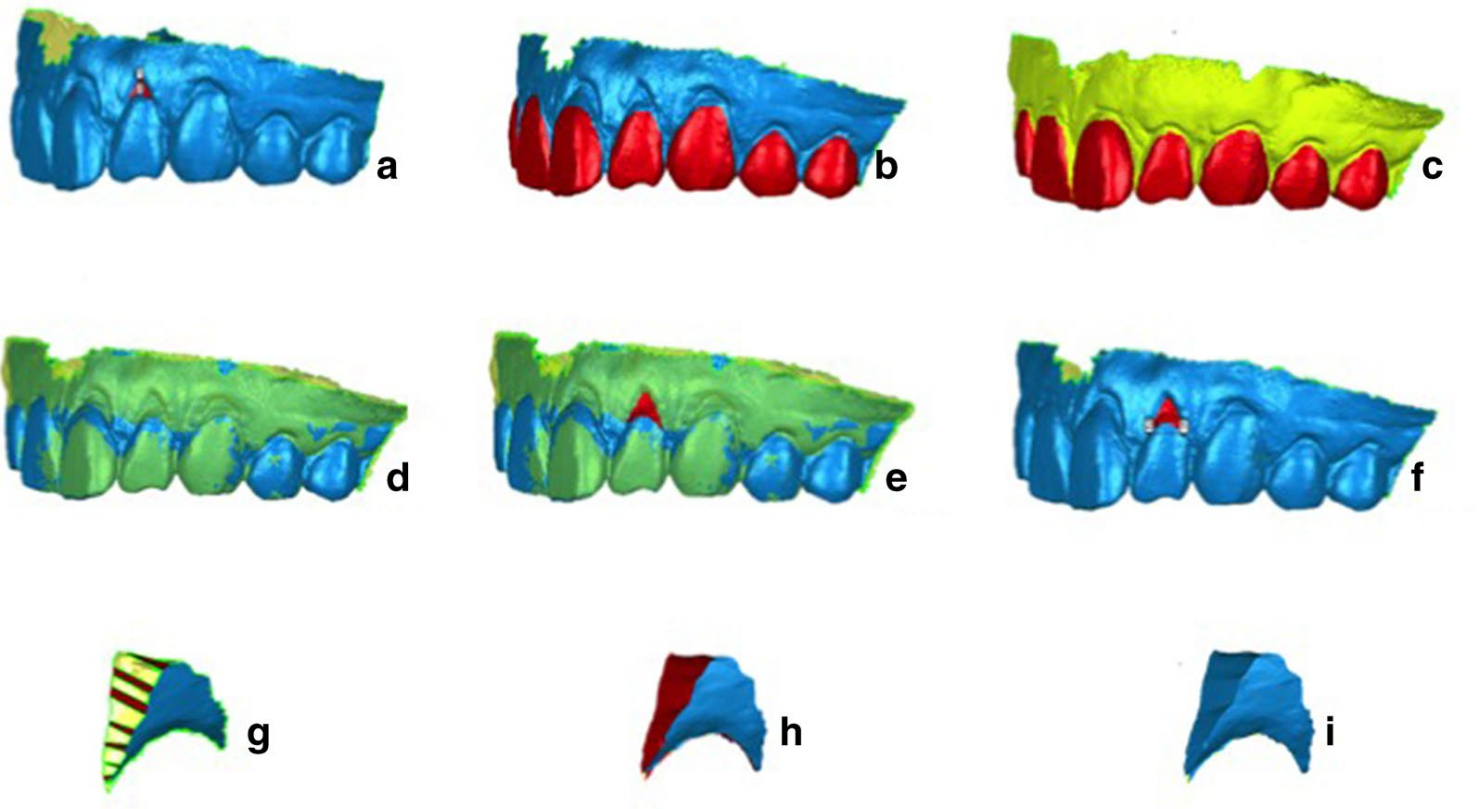
Three-dimensional digital measurement of the left upper lateral incisor between BL and 2w post-op. measurement of GRH, GRW, and REA on the BL model (**a**); similar surfaces of the teeth on the BL (**b**) and 2w (**c**) models were selected as the registration area (depicted in red); the two digital models overlapped for superimposition (**d**); manually trace of the postoperative gingival area gain (**e**); measurement of GHG and GAG on the BL model (**f**); closing the edges of the two digitized surfaces (**g**, **h**); reconstructed GVG (**i**)

Taking the left upper lateral incisor as an example (Fig. 2): Once superimposing the BL and translucent postoperative models, the range of ROI can be clearly distinguished. The GHG and GAG of the ROI were measured on the superimposed models. Then, a three-dimensional “crescent-shaped” ROI was reconstructed by closing the gap between the surfaces of the preoperative and postoperative model. The volume of the ROI was measured as GVG. GVG was then divided by the GAG to calculate the GMT of the ROI.

### Digital measurement of augmented buccal soft tissue

**Fig. 3:**
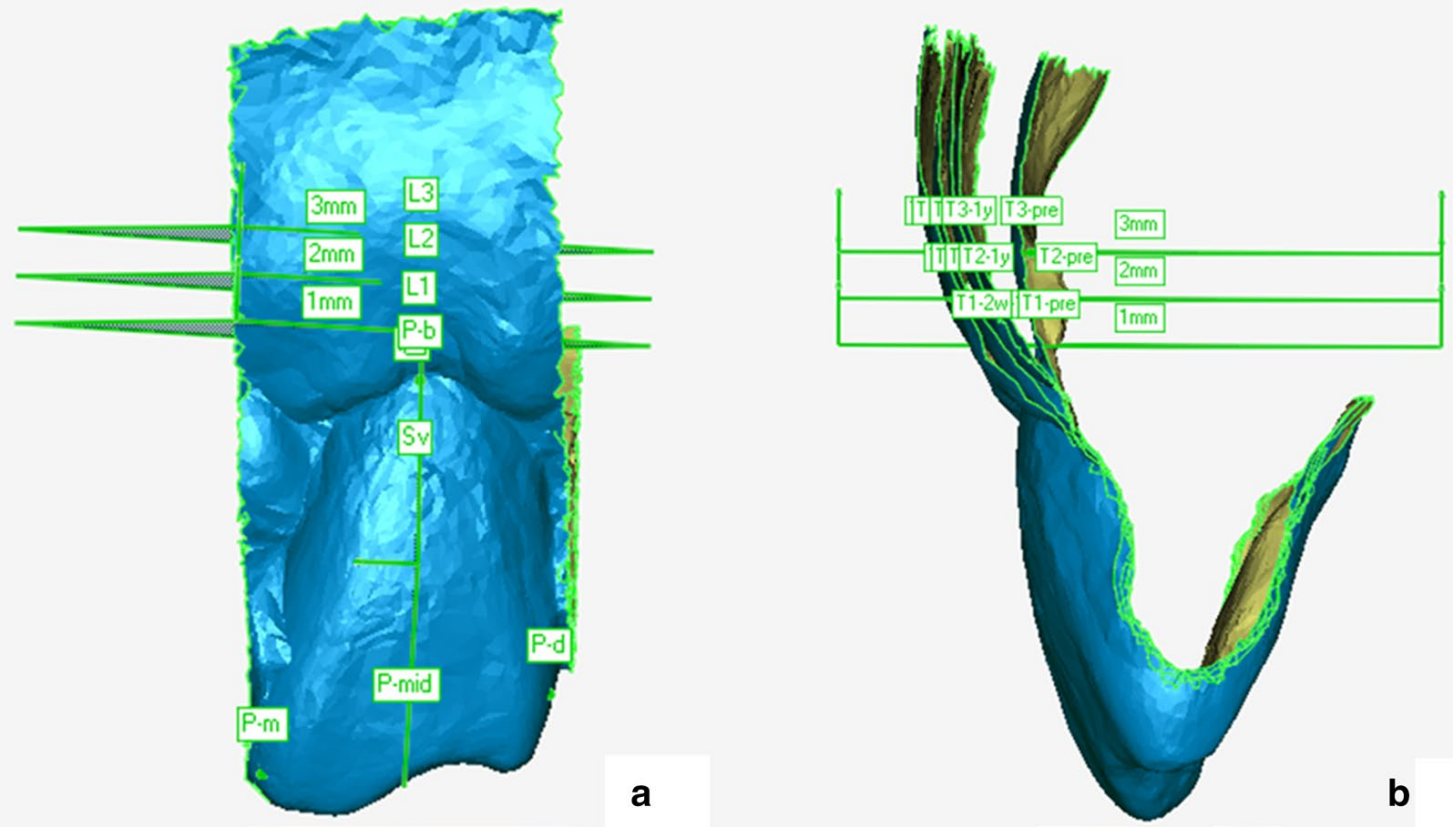
Frontal (**a**) and lateral (**b**) view of digital measurements of thickness alteration in buccal soft tissue

Digital measurements of dynamic thickness alterations in buccal soft tissue are shown in Fig. 3. The BL, 2w, 6w, 3m, and 1y digital models were superimposed together. The line L1 is perpendicular to the Z-axis on the sagittal plane Sv at the height of 1 mm apical to the CEJ. The points T1-2w, T1-6w, T1-3m, T1-1y, and T1-pre were obtained by intersecting line L1 with the corresponding digital models. The distance between points T1-2w and T1-pre was recognized as the tissue thickness change (TTC1-2w) at 1 mm apical to the CEJ 2 weeks post-op. TTC1, TTC2, and TTC3 represent the thickness changes of buccal tissue at 1, 2, and 3 mm apical to the CEJ. The remaining measurements were achieved in the same way.

### Statistical analysis

The variables analyzed were expressed as mean ± standard deviation (SD). Normality was checked using the Shapiro–Wilk test. Friedman’s test with Dunn’s multiple comparison correction was used to evaluate the difference of three-dimensional morphology changes in the ROI and buccal soft tissue over time. *p* < 0.05 was considered to indicate statistically significance.

## Results

A total of 25 teeth (13 in maxillary, 12 in mandibular) were included from 10 patients (5 males, 5 females) aged 26–53 years (average 36.13 ± 8.71 years). The KTW of these teeth at baseline was 2.565 ± 0.843 mm. All patients completed surgery and follow-ups, and no patients were dropped during the follow-up period. 22 of 25 teeth achieved complete root coverage at 1 year after MCAT with DGG surgery.

**Fig. 4:**
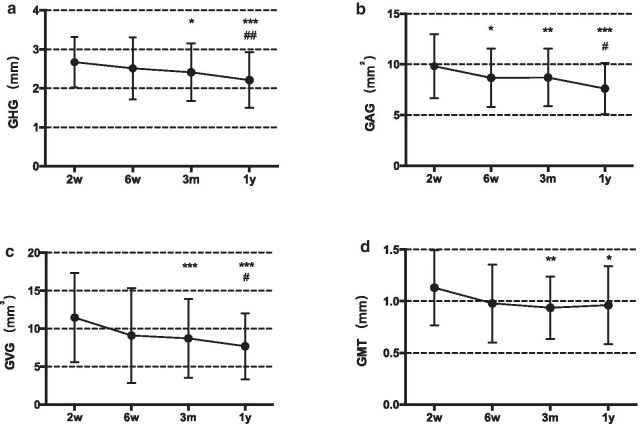
ROI volumetric dynamic alteration between 2 weeks and 1 year after the MCAT with DGG surgery. *p < 0.05, **p < 0.01, ***p < 0.001 indicate statistically significance compared to 2w. ^#^ *p* < 0.05, ^##^*p* < 0.01 indicate statistically significance compared to 6w

GRH, GRW and REA were 2.287 ± 0.521 mm, 2.907 ± 0.582 mm and 6.367 ± 1.634 mm^2^, respectively. The volumetric dynamic alteration of the ROI between 2 weeks and 1 year after the MCAT with DGG surgery are presented in Fig. 4; Table 1. Postoperative GHG, GAG, GVG, and GMT were encountered distinctly at 2w post-op, then gradually decreased. Statistically significant differences were recorded from 2w to 3m, 2w–1y and 6w–1y in terms of GHG, GAG and GVG. At 1 year, GHG, GAG, GVG and GMT were 2.211 ± 0.717 mm, 7.614 ± 2.511 mm^2^, 7.690 ± 4.335 mm^3^, and 0.965 ± 0.372 mm, respectively. The improvement in GMT after 6w was maintained with an increase of nearly 1 mm.

**Table 1:** ROI volumetric dynamic alteration between 2 weeks and 1 year after MCAT with DGG surgery

	2w	6w	3m	1y
GHG (mm)	2.672 ± 0.650	2.531 ± 0.793	2.411 ± 0.736*	2.211 ± 0.717*** ^##^
GAG (mm^2^)	9.823 ± 3.172	8.684 ± 2.871*	8.711 ± 2.856**	7.614 ± 2.511*** ^#^
GVG (mm^3^)	11.466 ± 5.895	9.101 ± 6.263	8.718 ± 5.197***	7.690 ± 4.335*** ^#^
GMT (mm)	1.131 ± 0.363	0.979 ± 0.378	0.936 ± 0.303**	0.965 ± 0.372*

*p < 0.05, **p < 0.01, ***p < 0.001 indicate statistical significance compared to 2w

^#^*p* < 0.05, ^##^*p* < 0.01 indicate statistical significance compared to 6w

**Fig. 5:**
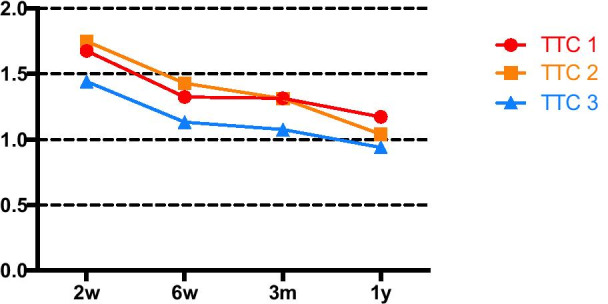
Dynamic alteration of buccal soft tissue thickness between 2 weeks and 1 year after the MCAT with DGG surgery. TTC1, TTC2, and TTC3 represent mean thickness change at 1, 2, and 3 mm apical to the CEJ

The dynamic alteration of buccal soft tissue thickness between 2 weeks and 1 year post-op is shown in Fig. 5. Similarly, the thickness of buccal soft tissue had the highest increase at 2w post-op, followed by a slightly reduction trend afterwards. The thickness change of buccal soft tissue 1 mm (TTC1) and 2 mm (TTC2) apical to the CEJ yielded higher results than those of 3 mm (TTC3).

## Discussion

The accuracy of the intraoral scanning systems has gradually improved in recent years. Not only can they replicate hard tissues such as teeth and restorations precisely, but they can also digitally reconstruct the surrounding soft tissues [20]. Some previous volumetric studies have use plaster models to analyze gingival dimension alteration after periodontal plastic surgery [21, 22]. However, recent surveys have revealed that the accuracy of intraoral scanning of soft tissue in the aesthetic area has reached or exceeded the capabilities of a plaster model [23, 24]. In addition, clinical researches have indicated that intraoral scanning method was superior to periodontal probe, plaster model, or scanning plaster model method when measuring the gingival recession and papillary height [15, 25]. In the present study, intraoral scanning was used to obtain digital models, which promise not only convenient and efficient operation, but also greater patient comfort, and convenient data storage. Combined with reverse engineering software, accurate, repeatable, and multi-dimensional measurement data can be obtained. In this study, REA, GAG, and GVG were measured using a digital method, which would have been impossible to achieve using traditional methods.

Previous studies have showed the soft tissue volume alterations qualitatively in chromatograms [16, 26]. However, the region defined by this method is too large and dependent upon the size of grafts. In contrast, the ROI in the present study may be more instructive for evaluation of the outcome of gingival augmentation and the relapse risk of gingival recession. The GMT of this study was lower than Rebele et al. using plaster models scanning [21]. This is likely because the ROI they had selected was 1 mm smaller than the ROI in the present study, discarding the surrounding thinner edges. However, it is generally stated that thin gingival phenotypes are at greater risk for developing gingival recessions than thick gingival phenotypes [27, 28]. This has been verified in studies of implants [29]. Therefore, the ROI depicted in this study may facilitate higher repeatability and play a more prominent role in assessing the long-term stability of the gingival margin.

The modified coronally advanced tunnel in combination with de-epithelialized gingival graft used in this study showed successful root coverage at 1 year post-op. This is consistent with other publications [7, 30], showing a high degree of root coverage of Miller I and II gingival recession. With the help of three-dimensional measurement, the present study found that GHG, GAG, GVG and GMT were all significantly higher at 2 weeks after surgery than baseline. All of these parameters decreased gradually over time, which was likely due to graft shrinkage and edema resolution. At 1 year post-surgery, GHG, GAG, and GVG of the ROI showed a significant reduction when compared to measurements taken at 2 weeks and 6 weeks. A significant decrease in GMT was only observed when compared to the 2 weeks measurements. However, no statistically difference was detected in any of the investigated parameters between 3 months and 1 year. This suggests a limited clinical impact in that the thickness of the ROI’s augmented tissue remained stable after 6 weeks post-op, while the volume maintained stability after 3 months post-op.

The present study proposes a new method for measuring the thickness change of buccal soft tissue, which is usually used to test the efficacy of the graft. By superimposing multiple digital models, more sophisticated data can be obtained. Since all of the treated sites achieved at least 3 mm of keratinized tissue post operation, the present study compared the thickness alteration of buccal soft tissue 3 mm apical to the CEJ. It was noted that the thickness change of buccal soft tissue was approximately 1 mm, which is comparable to data from previous studies [31, 6]. In addition, our study showed that the thickness change from 3 mm apical to the CEJ yielded inferior results than points closer to the gingival margin. This may have a beneficial effect on long-term stability of gingival margin.

However, the present study had several limitations. Firstly, the sample size was relatively limited and lacked a control group. In addition, whether the width of keratinized gingiva can be distinguished by color intraoral scan remains to be further studied. Nevertheless, the results of this study are valuable since we applied a reliable three-dimensional measurement protocol to detect volumetric alterations within 1 year after periodontal plastic surgery. Parameters of ERA, GAG, GVG and GMT, which would be difficult to measure accurately using traditional methods with periodontal probe, were able to be measured using this digital method. Furthermore, the present study was undertaken based on intraoral scanning, which will increase the validity of the data and enhance the quality of the study for its advantages of high reproducibility of results and small inter-examiner variation [15].

## Conclusions

Using intraoral scanning and analysis software, digital three-dimensional quantitative analysis is recommended to measure the dynamic alteration of buccal soft tissue after periodontal plastic surgery. Within the limitations of this study, outcomes indicated that buccal augmented tissue was stable after 3 months post-op and displayed only a slightly volumetric decrease over a follow-up period of 1 year post-op. Further clinical research is needed to determine what can make periodontal plastic surgery outcomes more predictable.

## Data Availability

The complete documentation of all patients enrolled in this study belongs to the authors and is available from Dr. Fei Xue only upon reasonable request.

## Abbreviations

CEJ: Cemento-enamel junction
MCAT: Modified coronally advanced tunnel
DGG: De-epithelialized gingival graft
MRC: Mean root coverage
CRC: Complete root coverage
RES: Root coverage esthetic score
SEI: Smile esthetic index
KTW: Keratinized tissue width
BL: Baseline
2w: 2 Weeks
6w: 6 Weeks
3m: 3 Months
1y: 1 Year
ROI: Region of interest
GRH: Gingival recession height
GRW: Gingival recession width
REA: Root exposure area
GHG: Gingival height gain
GAG: Gingival area gain
GVG: Gingival volume gain
GMT: Gingival mean thickness
TTC: Tissue thickness change
SD: Standard deviation
ANOVA: Analysis of variance
